# East Asian Winter Monsoon Impacts the ENSO-related Teleconnections and North American Seasonal Air Temperature Prediction

**DOI:** 10.1038/s41598-018-24552-3

**Published:** 2018-04-25

**Authors:** Tianjiao Ma, Wen Chen, Debashis Nath, Hans-F. Graf, Lin Wang, Jingliang Huangfu

**Affiliations:** 1 Center for Monsoon System Research, Institute of Atmospheric Physics, Chinese Academy of Sciences, Beijing, 100190 China; 2 School of Earth Science, University of Chinese Academy of Sciences, Beijing, 100049 China

## Abstract

El Niño-Southern Oscillation (ENSO) is a key feature for seasonal weather and climate prediction in the extra-tropics since related sea surface temperature anomalies induce precipitation anomalies that generate poleward propagating Rossby waves and teleconnections. The East Asian winter monsoon (EAWM) is driven by processes originating over the Asian continent and, to a lesser degree, by ENSO-related tropical convection. EAWM also strongly affects convection and precipitation patterns over the western tropical Pacific by cold air outbreaks reaching equatorial latitudes. Hence, one can expect a modulating effect of EAWM on the generation of Rossby wave trains related to ENSO. By increasing the convective heating over the western Pacific, strong EAWM strengthens the Pacific Walker circulation, and weakens (strengthens) the El Niño (La Niña) related effects on the extra-tropics via a modulation of the Pacific North America teleconnection pattern. Our results indicate that, for seasonal prediction over North America, along with ENSO the variability of EAWM should also be taken into account. The climate anomalies over the North America for the same phase of ENSO are significantly different for strong and weak EAWM.

## Introduction

**Figure 1:**
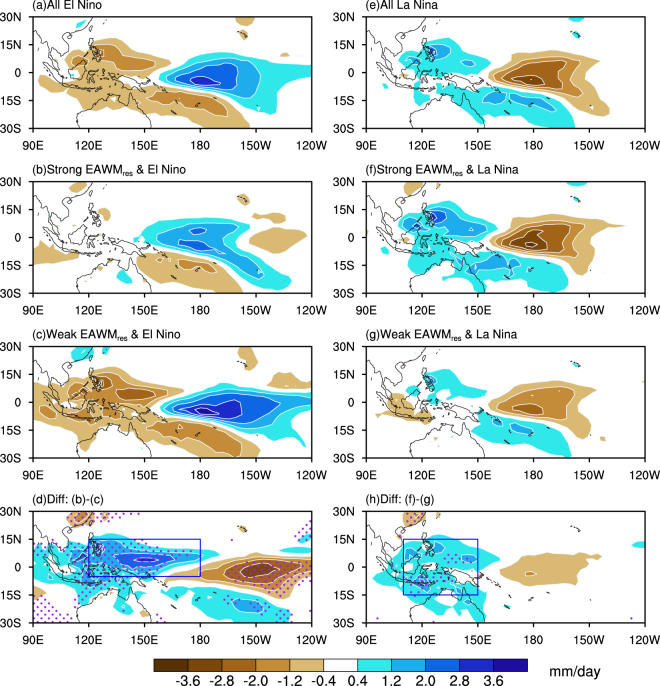
Composite winter mean (DJF) rainfall anomalies (Units: mm/day) in groups of: (**a**) mix of all El Niño events; (**b**) strong EAWM_res_-El Niño; (**c**) weak EAWM_res_-El Niño; (**d**) the difference between (**b**) and (**c**); (**e**) mix of all La Niña events; (**f**) strong EAWM_res_-La Niña; (**g**) weak EAWM_res_-La Niña; and (**h**) the difference between (**f**) and (**g**). Regions shaded with purple dots in (**d**) and (**h**) indicate the 90% confidence level. The maps in the figure are generated using the NCAR Command Language (NCL) (Version 6.4.0 & URL: http://www.ncl.ucar.edu/Download/).

El Niño-Southern Oscillation (ENSO) is the dominant interannual mode of climate variability in the tropical Pacific Ocean^1^. During an El Niño event, increased rainfall and intensified convection dominates the tropical central-eastern Pacific, while dry conditions prevail in the tropical western Pacific^2^ (Fig. 1a). The resulting anomalous diabatic forcing of the atmosphere, associated with ENSO, can affect the mid and high latitudes via atmospheric teleconnections^3–7^. This makes ENSO the most important predictor for global seasonal climatic anomalies^8^.

The East Asian winter monsoon (EAWM) system is characterized by a cold-core Siberian High, a warm-core Aleutian Low, and a predominant low-level northeasterly wind blowing from East Asia to the low latitudes in the western Pacific Ocean^9,10^. The EAWM can cause deep convection over the Maritime Continent, due to the intrusion of cold air into the tropics^11^, which is even linked to the Australian summer monsoon^12^. As a consequence, the East Asian Hadley circulation and the Walker circulations (the Indian and the Pacific Ocean cells) are intensified by the enhanced convective activity associated with the cold air outbreak events^13,14^. The EAWM links interannual climatic anomalies from the mid and high latitudes of the East Asian region to the tropical western Pacific Ocean^14^ which is one of the key heating centers driving the tropical atmospheric circulations. On the other hand, ENSO strongly impacts tropical climate variability at the interannual time scale. Therefore, it is of great interest to understand what degree the EAWM modulates the ENSO-related atmospheric forcing along the equatorial Pacific Ocean and how this may affect the extra-tropical weather and climate.

There are suggestions that ENSO has an influence on EAWM^15,16^, but the statistical evidence is weak (Table 1 shows roughly equal numbers of strong and weak EAWM for both, El Niño and La Niña, see also ref.^17,18^). The influence of the EAWM on ENSO at an interannual time scale so far has received much less attention. To evaluate the ENSO independent variability of the EAWM system, we follow the methodology proposed by Chen *et al*.^17^. They separate the variability of the EAWM into two parts: the EAWM_EN_ and the EAWM_res_. The EAWM_EN_ represents the variability of the EAWM which is correlated with the phases of ENSO. The EAWM_res_ is the ENSO-uncorrelated residual part, arising mainly from the internal processes of EAWM and the mid and high latitude processes over the Asian landmass. The EAWM_res_ and EAWM_EN_ accounts for ~87% and 13% of the total EAWM variability, respectively, which clearly indicates the dominance of the extratropical processes. Corresponding to a weak EAWM_res_, anomalous low-level circulations are featured by decreased northerlies over East Asia, a weakened Aleutian low and an enhanced western North Pacific anticyclone^17^. It should be noted that the linear regression technique cannot fully separate the independent influences of ENSO and the EAWM. However, the linear regression is a common approach in many of the previous studies^19,20^, and generally works because of little sea surface temperature (SST) anomalies in the tropics related to the EAWM_res_^17^.

**Table 1:** Distribution of the ENSO events based on the EAWM_res_, Cold Air Outbreak (CAO) frequency (times/winter) and interannual CAO strength index (normalized) in each group.

Groups	years	CAO Frequency	CAO Strength
Strong EAWM_res_-El Niño	[1953 1970^EP^ 1977^EP^ 1978^CP^ 1987^EP^ 2015^CP^]	5.8^**^	0.6^**^
Weak EAWM_res_-El Niño	[1954 1959^CP^ 1964 ^CP^ 1969^CP^ 1980 ^CP^ 1998^EP^ 2005 2010^CP^ 2016 ^EP^]	3.3^**^	−0.2^**^
Strong EAWM_res_-La Niña	[1956 1965^EP^ 1968^EP^ 1974^CP^ 1996^EP^ 1999^CP^ 2011^CP^]	5.3^*^	0.7^**^
Weak EAWM_res_-La Niña	[1950^EP^ 1972^CP^ 1975^EP^ 1976^EP^ 1985 ^EP^ 1989 ^CP^ 2001^CP^]	4.0^*^	−0.1^**^

Years 1953 indicate the winter mean of December 1952 to February 1953. The superscripts of EP (CP) indicate the Eastern (Central) Pacific type of ENSO. The double asterisk in the two El Niño groups (rows 1&2) denotes that the difference of CAO frequency (CAO strength) between the two El Niño groups exceeds the 95% (95%) one-tailed confidence level. The single (double) asterisk in the two La Niña groups (rows 3&4) indicates that the difference of CAO frequency (CAO strength) between the two groups of La Niña exceeds the 90% (95%) one-tailed confidence level, respectively.

The causal linkage between the ENSO events and interannual precipitation anomalies in the tropics is well reported^4,21^. However, the variability of tropical precipitation anomalies in the same phase of ENSO but under different extratropical circulation conditions is still not known. There is growing evidence that the extratropics can play an important role in ENSO evolution^22–25^. The primary objective of our present analysis is to investigate how and to what degree EAWM_res_ modulates the ENSO-SST related rainfall anomalies in the tropics. This study is of potential global significance, because the associated local or regional convective heating differences may trigger varied tropical-extratropical teleconnections due to poleward propagating Rossby waves being excited at different locations and with different strength.

Firstly, we present the composite rainfall anomalies over the Pacific Ocean under different background conditions as listed in Table 1 to show the modulating effect of the EAWM_res_ on the ENSO-related rainfall anomaly patterns in the tropical Pacific Ocean. Secondly, we illustrate the background atmospheric circulation conditions responsible for these rainfall anomalies over the Pacific Ocean. Thirdly, since the variability of ENSO has far-reaching impact on weather and climate over North America, we investigate how the EAWM_res_ is modulating the ENSO-related teleconnections and seasonal temperature anomaly patterns.

## Results

### SST related precipitation anomaly pattern modulated by EAWM_res_

The influence of EAWM_res_ on the ENSO-induced tropical rainfall anomalies during boreal winter months is shown in Fig. 1. The left panel displays the mean rainfall anomalies during El Niño conditions. A composite from 25 El Niño events, Fig. 1a, is plotted for the purpose of comparison. We observe an increase in rainfall over the central and eastern Pacific, while drier conditions prevail over the western Pacific. These results are consistent with the previous studies^4^. Next, we sorted the El Niño events into strong and weak EAWM_res_ conditions (Table 1). It should be noted that the subgroups of ENSO events have no bias against the ENSO intensity or spatial pattern changes. The intensity of SST anomalies in the Niño3.4 region (5°N-5°S, 120°–170°W) shows a roughly even distribution regardless of the EAWM_res_ status (see Section Methods and supplementary Figure S1). Furthermore, the two types of ENSO events^26,27^ have no obvious preference in any of the subgroups as shown in Table 1. It is also confirmed from the SST anomaly patterns for each of the four subgroups of ENSO that neither of them shows any consistent bias towards a central Pacific or an eastern Pacific type (supplementary Figure S2). SST anomalies in the weak EAWM_res_-El Niño are larger than those of the strong EAWM_res_-El Niño, but their differences are statistically insignificant. This could be attributed to the top 2 strongest El Niño events (1997/98, 2015/16) in the former subgroup.

The precipitation anomalies during the two subgroups of El Niño show obvious differences in amplitude and spatial extents. In the strong EAWM_res_-El Niño group, the below-normal (<= −1.2 mm/day) rainfall is restricted to small regions near the Philippines, and west of Indonesia, whereas for the weak EAWM_res_-El Niño group (Fig. 1c), drier conditions prevail over the entire western Pacific, the Maritime Continent and northern Australia. The daily average rainfall anomalies reach as low as −2.8 mm/day over the region between 0–15°N and 110°E–155°E. During the strong EAWM_res_-El Niño years, the eastern edge of the “wetter than normal” region in the northern hemisphere is confined to a region south of Hawaii in the central-eastern Pacific, while during the weak EAWM_res_-El Niño years it can extend further eastward to 120°W. The differences in rainfall anomalies between the strong and weak EAWM_res_-El Niño years are shown in Fig. 1d. Clearly the modulating effect of EAWM_res_ on the El Niño-related rainfall anomalies in the tropics can be observed. It is characterized by a positive center over the western and negative over the eastern Pacific Ocean. The anomaly ranges between ±2–3 mm/day, the same magnitude of mean El Niño-related anomalies. Hence, the EAWM_res_ is playing an important role in modulating the El Niño-induced rainfall anomalies over the tropical Pacific Ocean. The stronger the EAWM_res_ is, the weaker is the relationship between El Niño and tropical rainfall anomalies, and vice versa for weak EAWM_res_.

The right panel of Fig. 1 is similar, but for the La Niña conditions. Unlike for the El Niño conditions, the rainfall anomaly increases over the western Pacific Ocean, but decreases over the central equatorial Pacific Ocean (Fig. 1e). Strong EAWM_res_ under La Niña conditions brings heavier precipitation over the Philippines, the Maritime Continent and northern Australia (Fig. 1f). In contrast, weak EAWM_res_ under La Niña conditions favors abnormally low rainfall and drier conditions prevail in western Indonesia. The situation is very different over the central-eastern Pacific Ocean. Unlike during El Niño conditions, the EAWM_res_ couples only weakly with the tropical rainfall anomalies during La Niña conditions (Fig. 1h). On the one hand, this may be explained by the smaller amplitude of SST anomalies associated with La Niña. On the other hand, this may be attributed to La Niña related low SST over central-eastern Pacific and the threshold value (27–28 °C) for the occurrence of convection. Therefore, the EAWM_res_ has diverse influence on the ENSO-SST induced rainfall anomalies over the tropical Pacific Ocean.

The tropical rainfall anomaly patterns discussed above can be reconfirmed by several approaches. Firstly, as the subgroups have small numbers of samples for the composite analysis, we reconfirm our results using other datasets. The ECMWF’s 20th century (ERA20C) atmospheric reanalysis and Climatic Research Unit (CRU) timeseries (TS) version 4.00 precipitation data are employed for larger samples. The common time period in the two datasets is 1900–2010, and the years for each of the categories are listed in supplementary Table S1. Although limited by land precipitation only, results from these two longer datasets (supplementary Figure S3) are consistent with Fig. 1. Negative rainfall anomalies over the majority of the Maritime Continent during an El Niño is diminished by a strong EAWM_res_ and enhanced by a weak EAWM_res_. Similarly, Borneo, east Sumatra, and Java experience more rainfall during strong EAWM_res_-La Niña condition compared to that in weak EAWM_res_-La Niña condition. Secondly, results from the NOAA 20^th^ Century reanalysis datasets are largely consistent with the former ones (supplementary Figure S4 and Table S2). Note that supplementary Figure S4 shows little significance over the central equatorial Pacific Ocean, and this could be attributed to the uncertainty in the reconstructed precipitation dataset which have large ensemble spread over the tropical central Pacific Ocean. Thirdly, we employed different EAWM indices^28,29^ to get the ENSO-unrelated residual EAWM part. The variability of these “EAWM_res_” yields very similar rainfall anomaly patterns as presented in Fig. 1 (figures not shown).

### Possible mechanism

**Figure 2:**
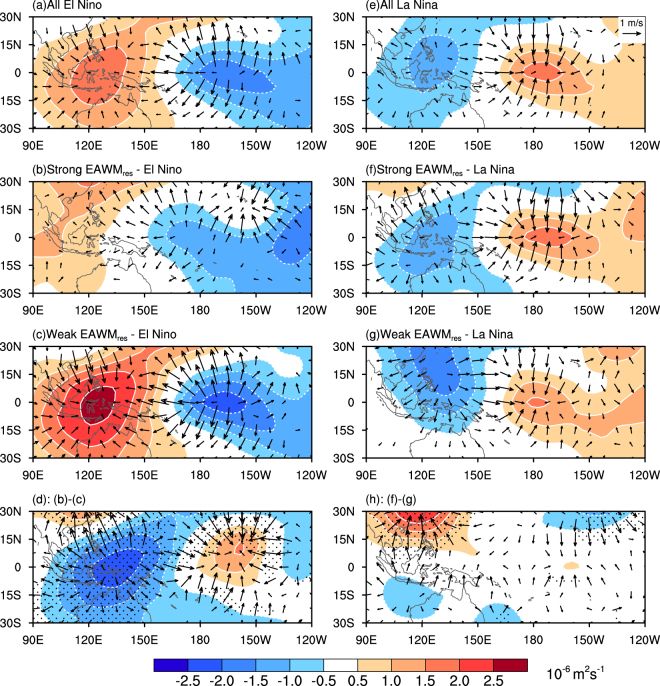
As in Fig. 1, but for 200-hPa divergent winds (vectors; m s^−1^) and velocity potential (contours; 10^−6^ m^2^ s^−1^). Regions shaded with black dots in (**d**) and (**h**) indicate that either the divergent winds or velocity potential are significant at the 90% confidence level. The maps in the figure are generated using the NCAR Command Language (NCL) (Version 6.4.0 & URL: http://www.ncl.ucar.edu/Download/).

The Indo-Pacific Walker circulation is affected by both ENSO- and EAWM-related rainfall anomalies^30^. In Fig. 2, anomalies of the Pacific Walker circulation are shown for all ENSO events and the different combinations of ENSO and EAWM_res_ (Table 1) as anomalies of the 200hPa divergent wind and velocity potential. The variation of the Pacific Walker cell is closely tied to the zonal SST gradient anomalies between the central and western equatorial Pacific. However, it is noteworthy that the difference of zonal equatorial Pacific SST gradient in the two El Niño (La Niña) subgroups is statistically insignificant (see section Methods). Climatologically, during an El Niño event, the weaker zonal thermal and pressure gradients weaken the Walker cell (Fig. 2a), as seen by anomalous ascending motion over the central-eastern Pacific Ocean and descending motion over the Maritime Continent (Fig. 2a). Stronger EAWM_res_ tends to weaken the Walker circulation anomalies initiated by the El Niño (Fig. 2b) i.e. convergence over the Maritime Continent has weakened to half the strength of its counterpart in the El Niño case. Figure 2c reflects the combined effect of weak EAWM_res_ and the El Niño, resulting in a much stronger western-Pacific convergence and eastern-Pacific divergence. During the La Niña episodes (Fig. 2e), the anomalous Walker circulation behaves contrastingly, with anomalous ascending (descending) motions over the tropical western (central) Pacific Ocean. The divergent center over western Pacific extends southwestward during the strong EAWM_res_-La Niña case, whereas, it shrinks northward during the weak EAWM_res_-La Niña case. As a consequence, convergence of air masses over the tropical central-eastern Pacific is strengthened (weakened) by stronger (weaker) EAWM_res_. It should be noted that the difference of Walker circulation anomalies between the strong and weak EAWM_res_ cases is weak during the La Niña events (Fig. 2h). This could be attributed to the suppressed convections in the central-eastern Pacific Ocean during a La Niña event. In other words, La Niña-related negative SST anomalies superimposed on the climatological cold tongue would greatly decrease the occurrence of deep convection. That means the EAWM_res_ would have limited impacts on tropical central-eastern Pacific during a La Niña event. What’s more, the smaller amplitude of SST anomalies associated with La Niña may also explain the weak difference of the Walker cell. In general, the variability of the Walker Circulation shown in Fig. 2 is resulting in the aforementioned rainfall anomalies including the asymmetry between the El Niño and La Niña events (Fig. 1).

Previous studies^13,14^ reported that the cold air outbreaks (hereafter CAOs, also called cold surges) can trigger deep convective activity in the tropics. The CAOs are dominant feature of EAWM, which usually start with an intensification of Siberian-Mongolian High. Vigorous northerlies along the eastern flank of the Siberian-Mongolian high lead to a sharp rise in sea level pressure and sudden drop in surface temperature over East Asian region. When the CAO progresses rapidly southward, a frontal system of high pressure and near surface strong northerly winds affects the tropics. These induce low-level convergence and cyclonic vorticity over the tropical western Pacific sea surface. Hence, vertical lifting associated with the low-level convergence and cyclonic anomalies contribute to deep convection and heavy rainfall during the penetration of CAOs^11^. Diabatic heating associated with the precipitation further accelerates both the local East Asian Hadley cell and the Walker cells over the Indian and Pacific Ocean^14^. Therefore, the mid-high latitude originated CAOs could affect the convections over tropics.

**Figure 3:**
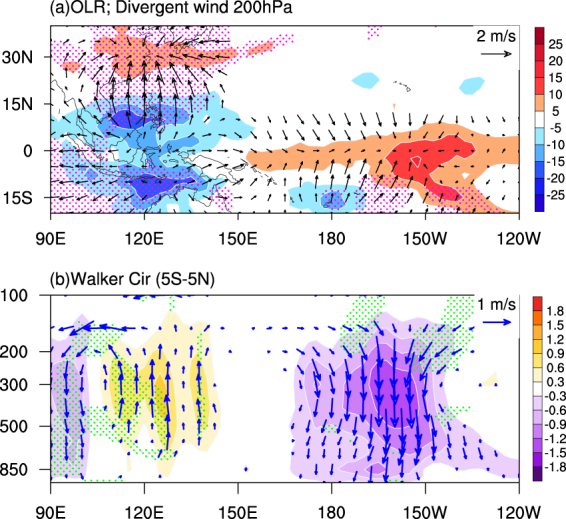
(**a**) Difference between strong and weak EAWM_res_ during the ENSO years: Outgoing Longwave Radiation (OLR; color filling, unit: W/m^2^); divergent winds at 200hPa (vector). The time period is 1979–2016 due to the short coverage of OLR dataset. (**b**) Difference of tropical omega (color filling) and zonal Walker circulation (vector) for average of 5°S-5°N between strong and weak EAWM_res_ during the ENSO years. The original omega (unit: Pa/s) is multiplied by −100.0 so that positive values indicate upward motion. Regions shaded with dots indicate the 90% confidence level. The maps in the figure are generated using the NCAR Command Language (NCL) (Version 6.4.0 & URL: http://www.ncl.ucar.edu/Download/).

We plotted the outgoing longwave radiation (OLR) in Fig. 3a as a proxy of tropical Pacific convective activity associated with the EAWM_res_. Due to the short time coverage of OLR dataset (1979–2016), we present the composite differences between strong and weak EAWM_res_ during all ENSO years without separating further into El Niño or La Niña cases. As the weak EAWM_res_ group contains more El Niño than La Niña events, weighted average is used to remove an El Niño bias. Similar averaging is performed for the strong EAWM_res_ group prior to the analysis. Clearly, strong EAWM_res_ favors enhanced convective activity over the tropical western Pacific and suppressed convective activity over the eastern Pacific Ocean (around 150°W). In Table 1 the frequency and strength of the cold air outbreaks in each of the subgroups is listed, indicating that strong EAWM_res_ intensifies the CAO activity, and vice versa during weak EAWM_res_. Thus, stronger EAWM_res_ with intensified CAO activity triggers enhanced convective activity over the Maritime Continent. The related diabatic heating anomalies associated with convection are the key to the Pacific Walker circulation variability (Fig. 3b). In general, strong EAWM_res_ triggers above-normal convective heating anomalies over the western Pacific Ocean, enhancing the climatological Walker circulation. It counteracts the El Niño-induced weakened Walker circulation anomalies, but amplifies the La Niña-induced enhanced anomalies over the tropical Pacific Ocean. The situation is opposite but weaker during weak EAWM_res_, when the El Niño-induced weakened Walker circulation is amplified and the anomalies due to La Niña are suppressed.

**Figure 4:**
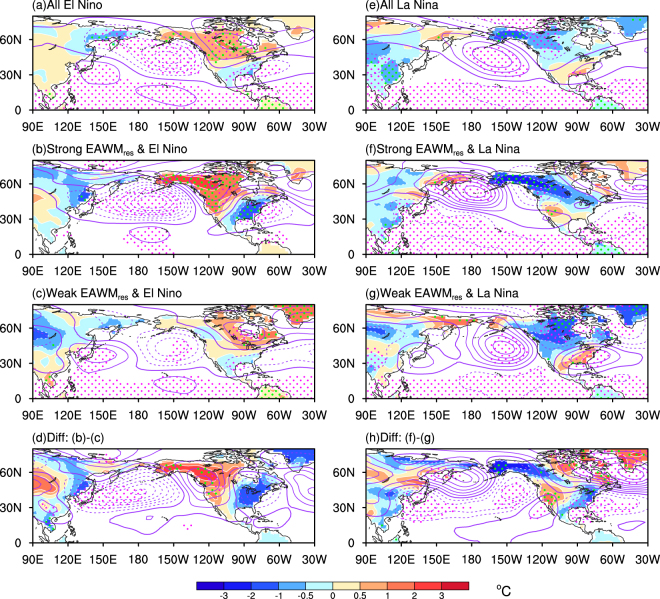
As in Fig. 1, but for winter-mean North American surface air temperature anomalies (color) and 500 hPa geopotential height anomalies (purple lines, CI = 10 gpm, zero line is bolded). Purple and green dots indicate that the HGT500 and SAT anomalies exceed 90% confidence levels, respectively. The maps in the figure are generated using the NCAR Command Language (NCL) (Version 6.4.0 & URL: http://www.ncl.ucar.edu/Download/).

The rainfall variability over the tropical western Pacific Ocean in response to ENSO was suggested to be directly related to the low-level circulation anomalies around the Philippines^15,16^. However, the cyclonic/anticyclonic flow over this region also has a close connection with the EAWM_res_. Feng and Chen^18^ reported that during an El Niño event, the Philippine anticyclone tends to be weak (strong) in presence of a strong (weak) EAWM_res_, and vice versa during the La Niña years (see ref.^18^ Figs 4, 5). Furthermore, according to the numerical experiments by Feng *et al*.^31^, during an El Niño event the negative convective heating anomaly around the Maritime Continent plays a dominant role for the existence of the Philippine anticyclone. Positive convective diabatic heating anomalies during strong EAWM_res_ balance the El Niño-related Maritime Continent negative heating anomalies, which weakens the Philippine anticyclone. On the other hand, convective cooling during weak EAWM_res_ strengthens the Philippine anticyclone, and vice versa for the La Niña. Thus, in the western Pacific the EAWM_res_ modulates the ENSO-related rainfall anomalies, mediated by the variation of low-level Philippine anticyclone.

The EAWM_res_ has diverse impacts on the ENSO-related tropical rainfall anomalies in the warm and cold phases: First, the EAWM_res_ has a weaker modulation effect during the La Niña than the El Niño events. Such asymmetric behavior is consistent with the fact that rainfall anomalies have a saturation effect in La Niña but more intense in the El Niño conditions^32^. Second, a stronger EAWM_res_ amplifies the rainfall anomalies over the region 5°S−15°N, 120°E–180°E under El Niño (Fig. 1d) and 15°S–15°N, 110°E–150°E under La Niña (Fig. 1h) conditions, respectively. The southwestward tilt in the precipitation anomaly pattern during La Niña conditions is in line with the trajectories of the cold air outbreaking events^16^. The Siberian High and the Aleutian Low experience a systematic eastward and westward shift in El Niño and in La Niña winters, respectively^16^. Hence, during El Niño cold air outbreaking events are more frequent across the Philippines. In contrast, during La Niña conditions cold air outbreaking events are shifted westward to the Sunda Strait^16^.

### Impacts on the US air temperature forecast

ENSO is reported to have significant impact on climate in the mid and high latitudes, particularly over the North America through the Pacific-North American (PNA) teleconnection pattern^33^. The longitudinal shift in the PNA pattern, due to the movement of the driving diabatic heating centers, is resulting in different climatic anomalies over North America^34^. Considering these complex interactions, it is interesting to investigate whether EAWM_res_ is modulating the ENSO-related climate anomalies (e.g. air temperature) over the North America. During the El Niño winter months, often the southern part of the United States (US) experiences colder than normal conditions, whereas the northern part of North American are warmer. The situation is just opposite during the La Niña winters (Fig. 4a and e). Taking also the information of EAWM_res_ into account, the North American air temperature anomalies display quite different patterns: the strong EAWM_res_-El Niño case is favoring a southeast (cold)–northwest (warm) contrast (Fig. 4b); weak EAWM_res_-El Niño initiates warmer conditions in northeastern North American. These variabilities in air temperature anomalies are consistent with the shift in the PNA-like pattern (Fig. 4, purple lines), which is generated by the latent heating centers in the tropics^27,34^. Stronger (Weaker) EAWM_res_, with central (eastern) Pacific latent heating (induced by above-normal rainfall anomalies, Fig. 1b and c), powers the westward (eastward) shift of the PNA-like pattern and the air temperature anomalies over the North America. Besides, the strength of Aleutian Low over North Pacific during the strong and weak EAWM_res_ – El Niño is related to the combined effect of El Niño and EAWM_res._ El Niño and strong EAWM_res_ contribute to an intensified Aleutian Low but weak EAWM_res_ acts opposite. Contrastingly, in the La Niña winters, the temperature difference between the two La Niña subgroups shows a quadrupole pattern, which is also consistent with the variation of 500 hPa geopotential height (Z500) (Fig. 4h). A wave train along Japan, west of Bering Sea, south to the Gulf of Alaska and eastern North Pacific is observed in Z500. The positive Z500 anomaly over Bering Sea and negative anomaly over Alaska favors stronger northerly winds between the two, which leads to a band of negative SAT over Alaska. Positive SAT anomalies over the western United States could be attributed to the southwesterly anomalies between the negative (north) and positive (southeast) Z500 anomalous centers over the eastern North Pacific. Results from a linear baroclinic model illustrated that the atmospheric linear response to an idealized western tropical Pacific thermal forcing is characterized by a wave train transporting from east of Japan to North America^35^. The wave train pattern in the North Pacific sector in Fig. 4h is similiar with the numerical results^35^, implying that the precipitation anomalies over the tropical western Pacific accounts for the different distributions of Z500 and SAT anomalies during two subgroups of La Niña. Surface air temperature differences between strong and weak EAWM_res_ for similar ENSO conditions can vary by 1–2°C, which is comparable to the anomalies due to ENSO alone. This indicates that EAWM_res_ is an additional important factor which may interfere with the seasonal North American air temperature prediction.

## Summary and Discussion

This study is mainly dedicated to a better understanding of the modulating effects of the EAWM_res_ on the ENSO-related rainfall and circulation variability in the tropics. Composite results suggest that a strong EAWM_res_ weakens the El Niño-induced Pacific rainfall and Walker circulation anomalies, while a weak EAWM_res_ amplifies them. A strong EAWM_res_ brings more cold air outbreaks to the low-latitudes. It intensifies the tropical deep convection and diabatic heating over the western Pacific. On the one hand, the anomalous heating weakens the low-level Philippine anticyclone and diminishes the consequent negative rainfall anomalies during the El Niño. On the other hand, the strong EAWM_res_ induced western Pacific heating, pumps the Pacific Walker circulation in a positive feedback loop. Over the eastern Pacific the stronger Pacific Walker circulation favors stronger surface divergence and less rainfall. Weak EAWM_res_, with below-normal heating anomalies over the western Pacific, will amplify the anomalies related to the El Niño. A similar EAWM_res_ intervention can be seen during the La Niña events. However, the EAWM_res_ appears to have a small contribution to the eastern Pacific rainfall due to the cold SST anomalies over there inhibiting convective rainfall anyway. Through its impact on West Pacific rainfall and the Pacific Walker cell, the EAWM_res_ also affects the ENSO-related temperature variability over the North America. Strong EAWM_res_ warms the western part and cools the eastern part of North America during an El Niño, whereas it cools the northwestern edge of North America and warms the western part of US during a La Niña. ENSO, thus, is not the only potential predictor for North American seasonal forecasts; the EAWM is similarly important and should be accounted for in the future.

The stratification of ENSO events into four subgroups lead to small sample sizes, and one may concern about the robustness of the composite. Hence, we add some supplementary analysis. As mentioned in results section (a), more samples (listed in supplementary Table S1) are obtained by using datasets which cover a longer period, the ERA20C atmospheric reanalysis and CRU data. Besides precipitation (supplementary Figure S3), composite anomalies of Walker cell and Z500, SAT (supplementary Figures S5, 6) are consistent with the former results in Figs 2 and 4, respectively.

## Methods

### Datasets

The National Centers for Environmental Prediction - National Center for Atmospheric Research (NCEP-NCAR) monthly mean atmospheric reanalysis data^36^ are used in this study. This datasets have a horizontal resolution of 2.5° latitudes by 2.5° longitudes, 17 levels in the vertical direction, and a time period of 1948-present. For sea surface temperature (SST) we used the 1.0° × 1.0° horizontal resolution monthly mean Hadley Centre Global Sea Ice and Sea Surface Temperature (HadISST) dataset^37^. The HadISST data covers the time period from 1870 to the present. In addition, for rainfall data we employed the globally gridded (2.5° × 2.5°) monthly-mean NOAA Precipitation Reconstruction (PREC) data from 1948 to the present^38^. The NOAA Outgoing Longwave Radiation (OLR)^39^ data, available from 1979 to 2016, is used to signal the activities of convection.

We use the data averaged from December to February to represent boreal winter mean. The analysis period spans from 1948 to 2016, except for that related with OLR (from 1979 to 2016). Conventionally, the winter of 1949 refers to the December 1948 to February 1949. All the original datasets are subjected to a 7-year high pass Lanczos filter to suppress the decadal variability. Moreover, we also use the monthly mean 20^th^ century reanalysis datasets^40^ (from 1851 to 2012), ERA-20C reanalysis datasets^41^ (from 1900 to 2010), Climate Research Unit (CRU) Time-Series (TS) version 4.00 gridded temperature and precipitation data^42^ (from 1901 to 2015), and the NOAA Extended Reconstructed Sea Surface Temperature (ERSST) V4 (1854-present)^43,44^ to compare our results.

### Indices and methods

The EAWM_res_ index^17^, which represents the interannual variability of EAWM_res_, is calculated by removing the ENSO-related part by linear regression. The original EAWM_res_ index is multiplied with −1, so that a positive index corresponds to northerly winds and a strong EAWM_res_ state. Strong (Weak) EAWM_res_ winters are selected based on a threshold of + (−) 0.5 standard deviations of EAWMI_res._ We then obtain 20 strong and 20 weak EAWM_res_ winters over the period of 1948–2016, respectively.

The Oceanic Niño index (ONI), which is defined by the Climate Prediction Center (CPC) as the 3-month running mean of ERAAT.v4 SST anomalies in the Niño-3.4 region (5°N–5°S, 120–170°W), is used as an ENSO index here. According to the CPC, when the ± 0.5°C threshold of ONI is met for a minimum of 5 consecutive overlapping months, it can be recognized as an El Niño/La Niña event. Based on these criteria, we obtain 25 El Niño and 20 La Niña events from the 69 winters between 1948 and 2016. ENSO events after 1950 are obtained from the CPC website http://www.cpc.ncep.noaa.gov. The ENSO events are further sorted into 4 groups based on different conditions of EAWM_res_.

1. Strong EAWM_res_ with El Niño
2. Weak EAWM_res_ with El Niño
3. Strong EAWM_res_ with La Niña
4. Weak EAWM_res_ with La Niña

All these selected years are listed in Table 1. It should be noted that the oceanic Niño index (ONI) is linearly independent of the EAWM_res_ index. And the intensity of ENSO events shows a roughly even distribution regardless of the EAWM_res_ status (supplementary Figure S1).

Eastern-Pacific (EP) and Central-Pacific (CP) ENSO events are identified by normalized winter mean Niño 3 index and El Niño Modoki index (EMI)^26^. When the normalized EMI exceeds 0.7 (−0.7), meanwhile greater (less) than the normalized Niño 3 index, a CP El Niño (La Niña) is defined. In the same way, an EP El Niño (La Niña) is identified when the normalized Niño 3 index greater (less) than 0.7 and also greater (less) than the EMI. Years that both of the two Niño indices don’t exceed a 0.7 standard deviation would not be classified to any type. Thus, two types of ENSO are separated as listed in Table 1.

The zonal SST gradient along the equatorial Pacific Ocean between the central and western Pacific is defined as the difference in area averaged SST between the Nino 4 (160°E-150°W, 5°S-5°N) region and the western Pacific Ocean (90°-150°E, 5°S-5°N). Result of 1000 bootstrapping^45^ combinations shows that the zonal SST gradient difference between the strong EAWM_res_-El Niño group and the weak EAWM_res_-El Niño group (the strong EAWM_res_-La Niña group and the weak EAWM_res_-La Niña group) is insignificant at the 90% confidence level based on a two-tailed test. The result is same if considering the zonal SST gradient between the eastern (Nino3 region, 150°W-90°W, 5°S-5°N) and the western equatorial Pacific.

### Cold Air Outbreak

The winter frequency of CAO events is obtained from Abdillah^46^ (Figure A2; W-CAO over East Asian; period 1958–2013). Table 1 listed the average winter frequency of East Asian CAO events in each group. As the samples sizes are small, we used the bootstrap method^45^, resampling 1000 times, and one-tailed test to estimate the mean differences from two samples. The winter frequency of CAO events in the strong EAWM_res_-El Niño group is significantly higher than that in the weak EAWM_res_-El Niño group at a confidence level of 95%. Between the two groups during the La Niña, the difference reaches the 90% confidence level.

We adopt an alternative approach to estimate the interannual variation of CAOs. A profound characteristic related with CAOs in the tropics is the disturbance of sea level pressure (SLP)^13,14^. Usually, the time scale of a CAO event is about 5 days^47^, therefore a 3–6 day band pass filter is applied on the SLP anomaly over the western tropical Pacific region to define the East Asian cold air outbreaking events. Thus, an annual CAO strength index is defined as an area-averaged variance of the filtered anomalies during winter in the region between 0–15°N and 105°E-125°E. We checked the robustness of the index by considering smaller and bigger areas, however, the result remains the same. The average strength of the CAO strength index (normalized) is −0.1 for El Niño (25 events) and 0.1 for La Niña (18 events) conditions, reflecting the influence of ENSO on cold surges^46^. But when we include the EAWM_res_ variability, the CAO strength index for the strong EAWM_res_ groups is much higher than for the weak EAWM_res_ groups. The values are 0.6&-0.2 for El Niño and 0.7&-0.1 for La Niña, respectively. The difference of CAO strength indices between the strong EAWM_res_- El Niño (La Niña) group and the weak EAWM_res_- El Niño (La Niña) group exceed the 95% (95%) confidence level according to a one-tail bootstrap statistical estimate. This indicates that strong EAWM_res_ intensify the CAO activities, and vice versa during weak EAWM_res_, regardless of El Niño or La Niña background.

### Divergent wind calculation

The horizontal wind can be divided into two components: non–divergent (or rotational) and divergent wind^48^.

1 $$\overrightarrow{V}=\overrightarrow{{V}_{\psi }}+\overrightarrow{{V}_{\chi }}$$

In equation (1), ${\rm{\psi }}$ represents stream function and ${\rm{\chi }}$ represents velocity potential.

2 $${u}_{\psi }=-\,\frac{\partial \psi }{\partial y},{v}_{\psi }=\frac{\partial \psi }{\partial x}$$

3 $${u}_{\chi }=\frac{\partial \chi }{\partial x},\,{v}_{\chi }=\frac{\partial \chi }{\partial y}$$

In equations (2) and (3), ${u}_{\psi }$, ${v}_{\psi }$ stand for the rotational components of horizontal wind and ${u}_{\chi }$, ${v}_{\chi }$ represent the divergent wind. The horizontal divergence (D) is calculated by

4 $${\rm{D}}=\,\nabla \cdot \overrightarrow{V}=\nabla \cdot \overrightarrow{{V}_{\psi }}+\nabla \cdot \overrightarrow{{V}_{\chi }}=\nabla \cdot \overrightarrow{{V}_{\chi }}$$

Hence, substituting equations (3) into (4) yields

5 $${\rm{D}}=\frac{{\partial }^{2}{\rm{\chi }}}{\partial {{\rm{x}}}^{2}}+\frac{{\partial }^{2}\chi }{\partial {y}^{2}}$$

Therefore, velocity potential ${\rm{\chi }}$ can be obtained by solving the Poisson equation according to the equation, then ${u}_{\chi }$, ${v}_{\chi }$ can be obtained via equation (3).

## Electronic supplementary material

Supplementary Information
