# Supplementary material for: East Asian Winter Monsoon Impacts the ENSO-related Teleconnections and North American Seasonal Air Temperature Prediction
Source: Sci Rep. 2018 Apr 25;8:6547. doi: 10.1038/s41598-018-24552-3 (PMC5917031; doi:10.1038/s41598-018-24552-3)
Supplement: Supplementary file 1 — Supplementary Information [file 41598_2018_24552_MOESM1_ESM.pdf]

Supplementary Information

East Asian Winter Monsoon Impacts the ENSO-related Teleconnections and North American Seasonal Air Temperature Prediction

Tianjiao Ma[1, 2], Wen Chen[1, 2*], Debashis Nath[1], Hans-F. Graf[1#], Lin Wang[1], and Jingliang Huangfu[1]

Affiliation:

[1] Center for Monsoon System Research, Institute of Atmospheric Physics, Chinese Academy of Sciences, Beijing 100190, China

[2] School of Earth Science, University of Chinese Academy of Sciences, Beijing 100049, China

[*]**Corresponding Author:**

Wen Chen
Institute of Atmospheric Physics
Chinese Academy of Sciences
Beijing 100190, China
Ph: +86 10 62551597
e-mail: cw@post.iap.ac.cn

[#] Visiting Fellow of the Chinese Academy of Sciences

Supplementary Tables

Table S1. Distribution of the ENSO events based on the $EAWM_{res}$ **with the ERA20C reanalysis datasets.** Years 1905 indicate the winter mean of December 1904 to February 1905. Bolded font marks the years identical with those in Table 1 for the overlapping period.

Groups	years
Strong $EAWM_{res}$-El Niño	[1905 1914 1926 1940 **1953 1970 1977 1978** 1988 1995]
Weak $EAWM_{res}$-El Niño	[1906 1915 1919 1924 1942 **1954 1959 1964 1969** 1973 **1998**]
Strong $EAWM_{res}$-La Niña	[1910 1918 1934 1951 1955 **1968** 1971 **1974 1996 1999**]
Weak $EAWM_{res}$-La Niña	[1909 1911 1916 1939 1950 **1972 1976 1985 1989 2001**]

Table S2. Same as Table S1, but with **the NOAA 20th century reanalysis datasets**.

Groups	years
Strong $EAWM_{res}$-El Niño	[1856 1881 1885 1886 1889 1905 1912 1914 1926 1940 1946 **1953** 1958 **1970 1978 1987** 1988 1995 2007]
Weak $EAWM_{res}$-El Niño	[1900 1906 1915 1919 1924 1931 1941 1952 **1954 1959 1969** 1973 **1980 1998** 2003]
Strong $EAWM_{res}$-La Niña	[1870 1873 1879 1893 1911 1917 1918 1934 1951 **1956 1968 1974 1999 2011**]
Weak $EAWM_{res}$-La Niña	[1857 1876 1880 1887 1890 1894 1909 1916 1921 1950 1965 **1972 1985 1989 2001**]

Supplementary Figures

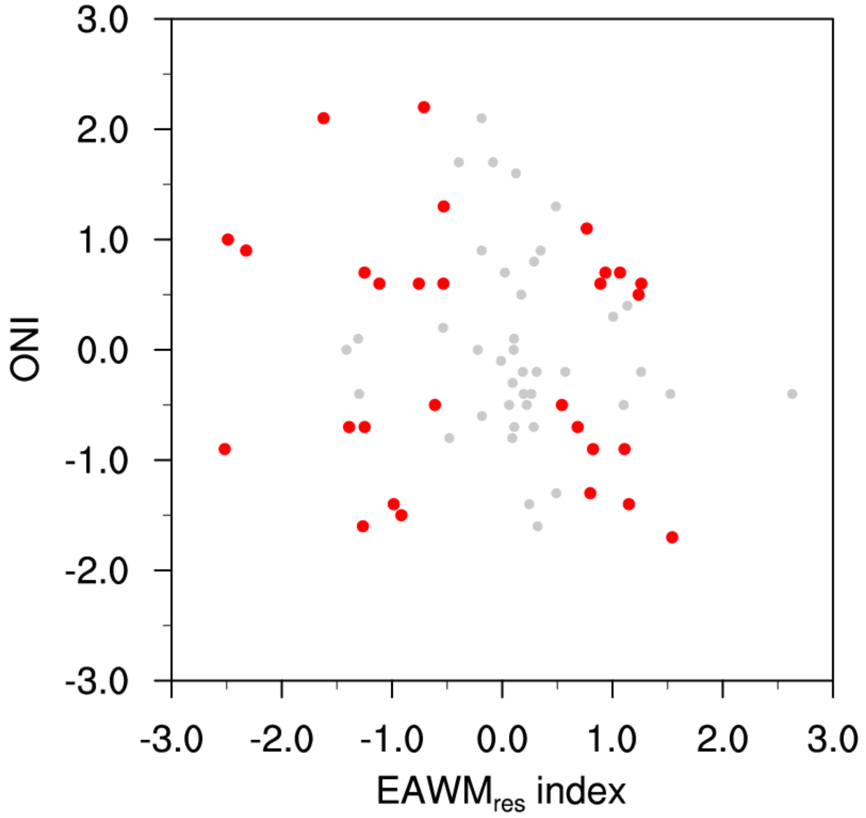

Figure S1. Distribution of Ocean Nino index (ONI, from NOAA Climate Prediction Center) based on EAWM$_{res}$ index (gray dots, a positive value of EAWM$_{res}$ index indicates a stronger-than-normal EAWM$_{res}$ winter). Red dots represent the selected ENSO winters in Table 1.

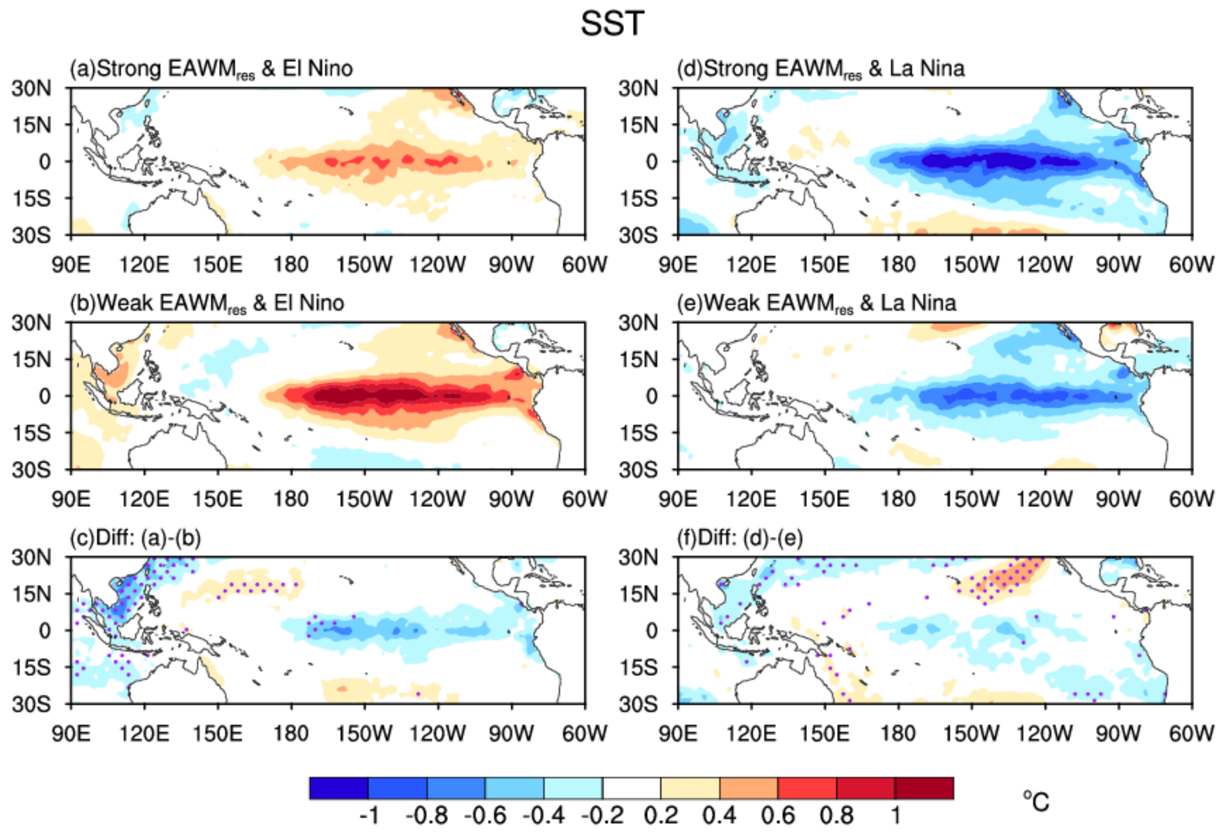

Figure S2. Composite wintertime (DJF) SST anomalies during strong $EAWM_{res}$–El Niño (a) and weak $EAWM_{res}$–El Niño winters (b). (c) is the difference between (a) and (b). (d), (e), and (f) is the same as (a), (b), and (c) but for La Nina groups. Dots in (c) and (f) indicate that the difference is significant at 90% confidence levels. The maps in the figure are generated using the NCAR Command Language (NCL) (Version 6.4.0 & URL: http://www.ncl.ucar.edu/Download/)

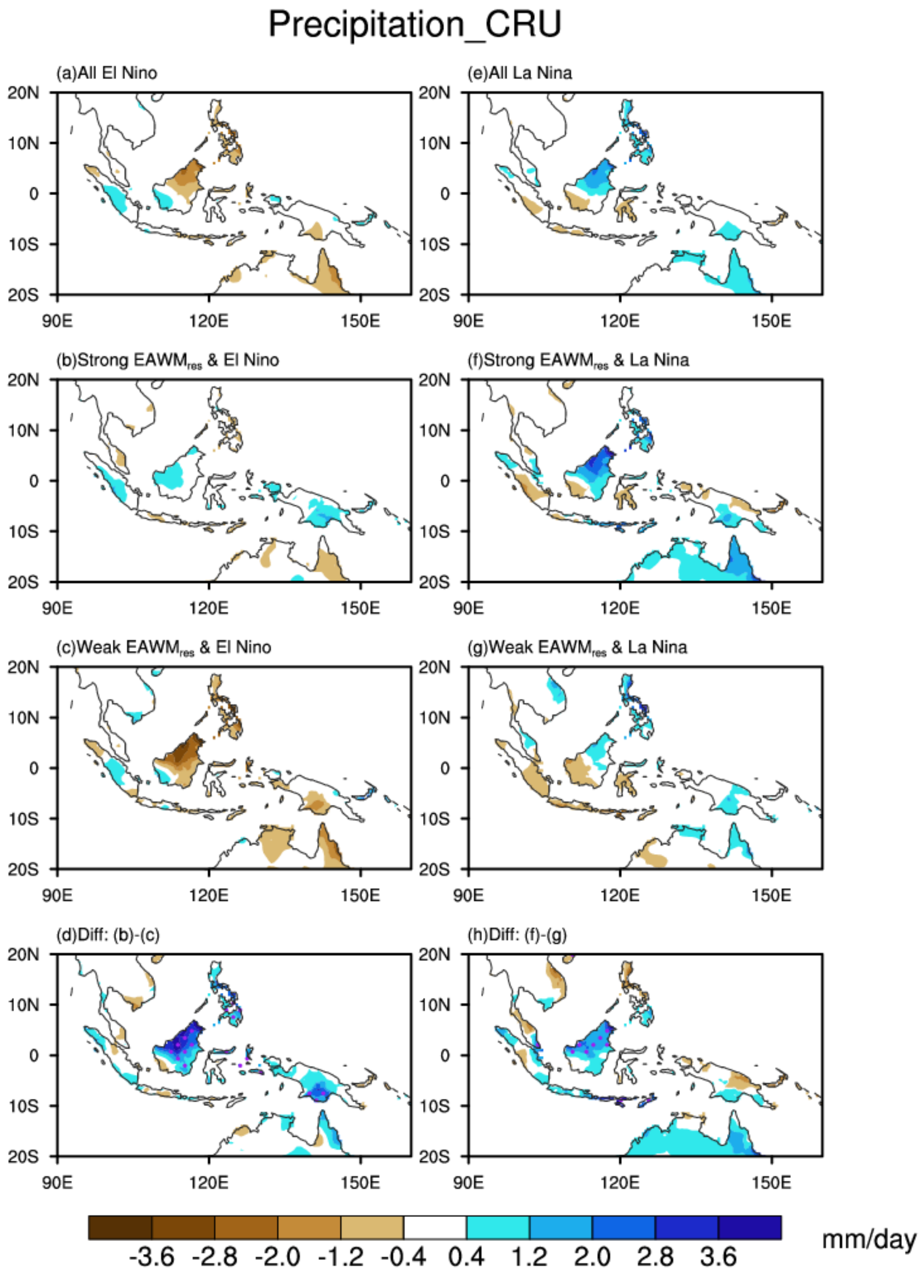

Figure S3. As Figure 1, but using Climate Research Unit (CRU) version 4.00 precipitation data (period: 1900-2010). Composite years for each category are listed in Table S1. Dots in (d) and (h) indicate that the difference is significant at 90% confidence levels. The maps in the figure are generated using the NCAR Command Language (NCL) (Version 6.4.0 & URL: http://www.ncl.ucar.edu/Download/)

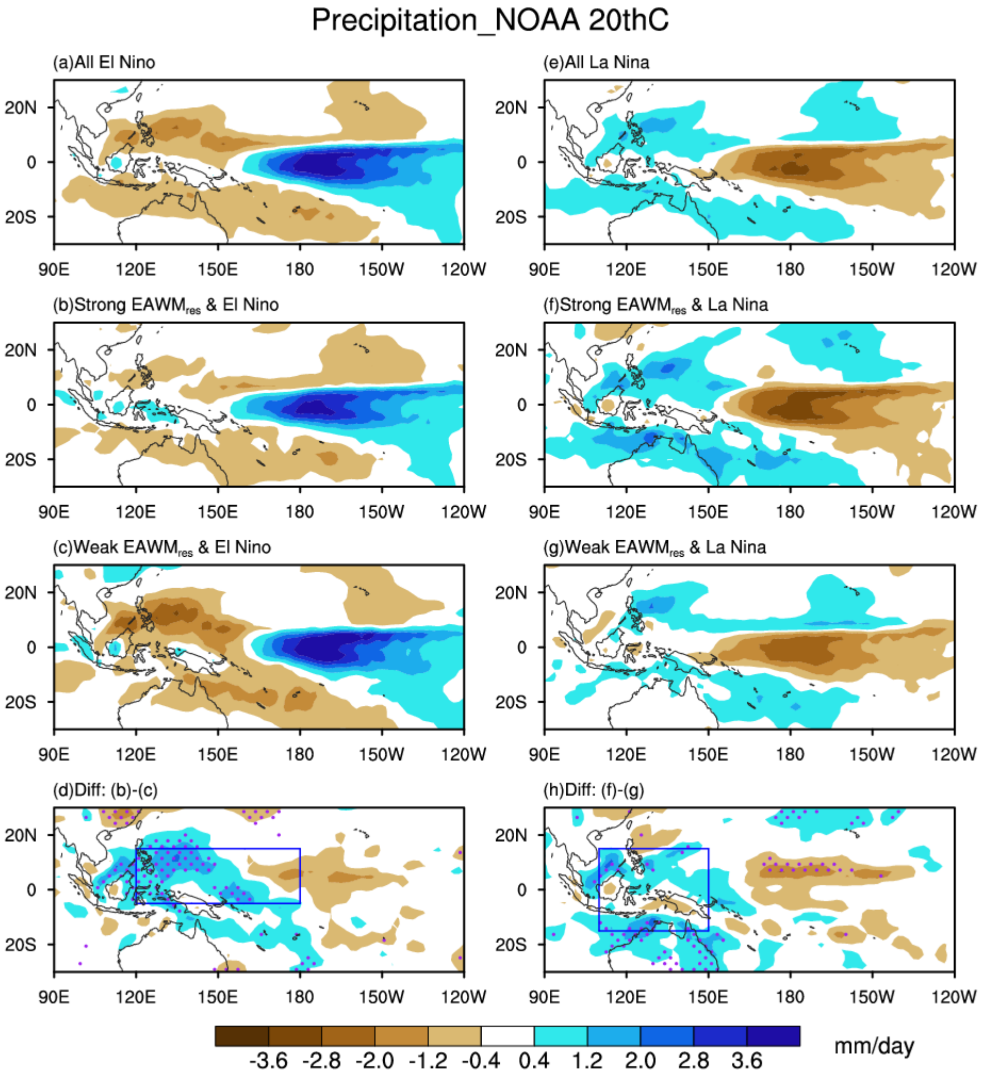

Figure S4: As Figure 1, but using NOAA 20th century reanalysis datasets for precipitation. The maps in the figure are generated using the NCAR Command Language (NCL) (Version 6.4.0 & URL: http://www.ncl.ucar.edu/Download/)

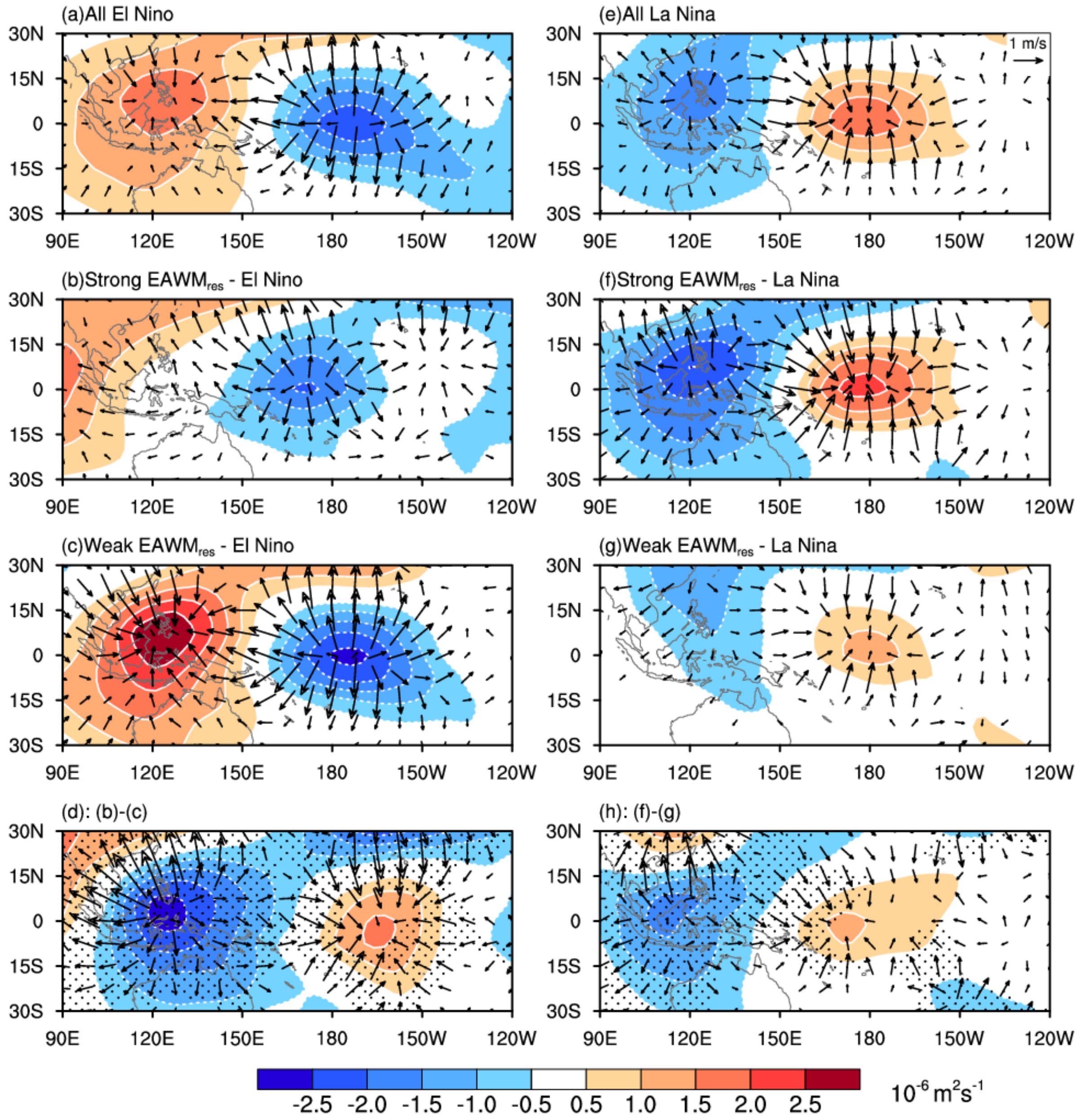

Figure S5: As Figure 2, but using ERA20C reanalysis datasets. The composite years for each category are listed in Table S1. The maps in the figure are generated using the NCAR Command Language (NCL) (Version 6.4.0 & URL: http://www.ncl.ucar.edu/Download/)

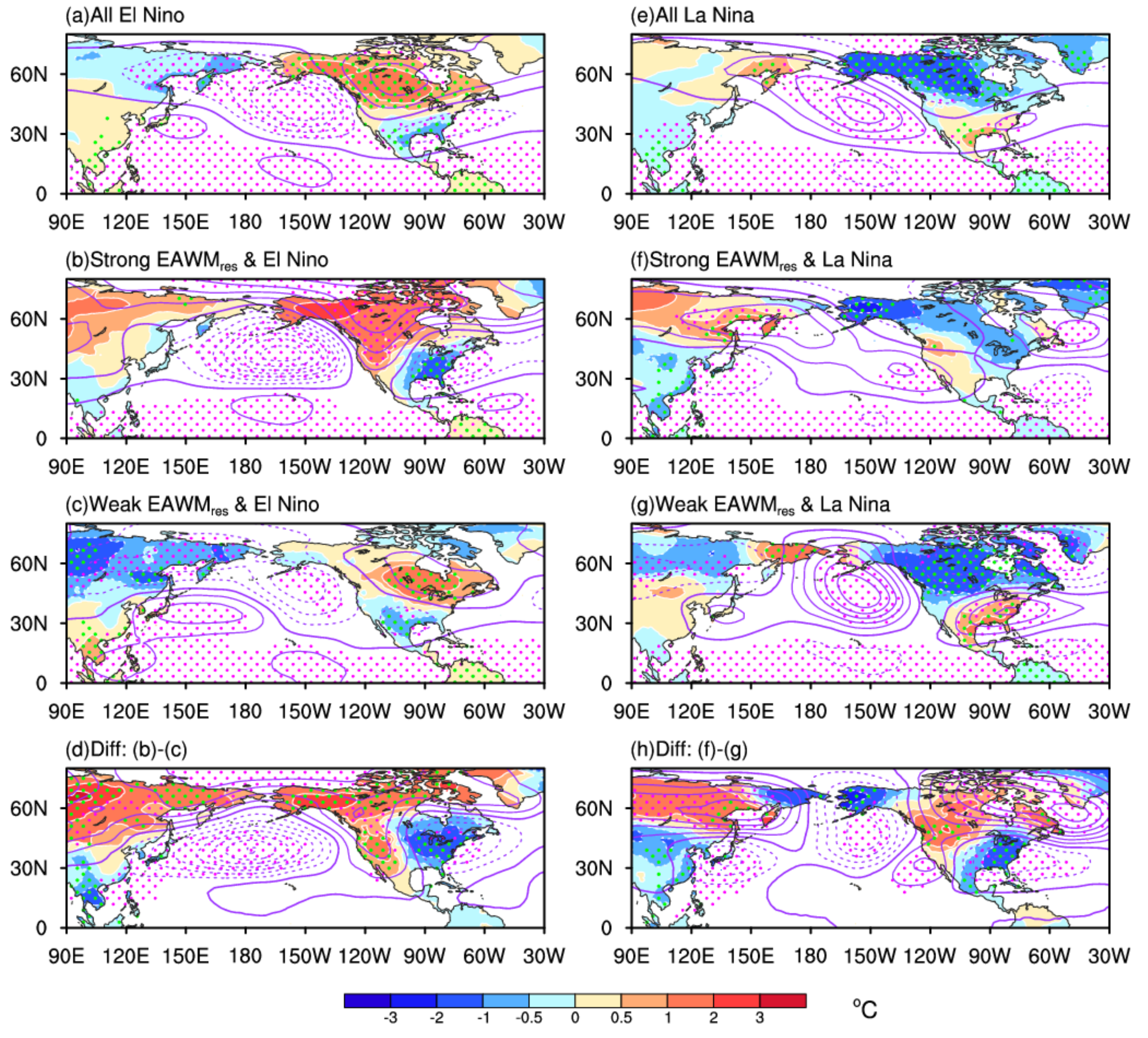

Figure S6. Same as Figure 4, but using CRU surface temperature data and ERA20C geopotential height data (period: 1900-2010). The composite years for each category are listed in Table S1. The maps in the figure are generated using the NCAR Command Language (NCL) (Version 6.4.0 & URL: http://www.ncl.ucar.edu/Download/)
